# Design Visual Elements and Brand-Based Equity: Mediating Role of Green Concept

**DOI:** 10.3389/fpsyg.2022.888164

**Published:** 2022-04-28

**Authors:** Ying Li

**Affiliations:** Creative Design College, Hainan Tropical Ocean University, Hainan, China

**Keywords:** design perception visual, design perception functional, design perception kinesthetic, green concept, employee-brand based equity

## Abstract

Although benefits of design perception have been documented from the perspective of consumers on a large scale, but the perspective of employees has been ignored. This study aims to investigate the impact of design elements on employee-brand-based equity under the mediating role of the green concept. For this purpose, data are collected from the employees of the manufacturing sector and 346 responses are used for an inferential purpose. These data were collected using the survey research method through the convenience sampling technique. Data has been analyzed through Smart PLS by applying the structural equation modeling technique. After assessing the measurement and structural model, the results obtained indicate that design elements of products in the manufacturing industry can influence the perception of employees and it can foster positive behavior among employees in the shape of employee-brand-based equity. The mediating role of the green concept has also been proved. Limitations and future directions are also discussed.

## Introduction

Benefits of design perception have been documented from the perspective of consumers at large, and functional design is studied extensively in the literature, most notably as utilitarian/functional benefits (Petruzzellis, 2010). It refers to the physical attributes of a product, including features, performance, dependability, and durability, that meet a user’s sanitary needs and expectations. These intangible elements are concealed from view by users, silently operating in the background to improve the user experience. From a means-end viewpoint, a product’s functional benefits should meet higher-order requirements. These intangible elements are concealed from view by users, silently operating in the background to improve the user experience. From a means-end viewpoint, a product’s functional benefits are the bare minimum that it must provide before being supplemented with other design aspects to meet higher-order requirements.

While no particular study has investigated that the design perception of products can also create a positive impact on the behavior of the employee. Design is a significant strategic instrument and literature provides support that it may improve product competitiveness but how it can develop positive behaviors in the workplace is still an unexplored area. Perception of visual design is a key factor in experiencing value. The external look of things translates into perceived usefulness *via* subtle clues concerning ease of use, which leads to the creation of attitudes about usability (Creusen and Schoormans, 2005). Furthermore, the visual design conveys a symbolic significance that influences aesthetic judgment, allowing the owner to utilize the object as a means of self-expression. The literature lacks sufficient studies that documented the impact of design perception in shaping positive behaviors in the workplace. Moreover, how a design perception can influence the green concept is an unexplored area of research, and researchers need to put efforts into identifying and resolving such issues because social and individual drivers have the potency to impact design perception (Hemonnet-Goujot and Valette-Florence, 2022).

From a theoretical perspective, this study is the first to document the impact of the element of design perception on employee-brand based equity (EBBE). This study adds to the literature stream of brand-based equity research and contends that design elements of products in the manufacturing industry can influence the employees’ perception. Additionally, this study also advocates that positive evaluation of design perception by the employees can foster positive behavior among employees. So this study can be termed the first study which has examined the relationship between design perception elements and EBBE. Second, this study tested the mediating role of the green concept, which is another contribution of this study. This study tends to advocate that the green concept can increase EBBE. There is a need to investigate non-financial elements which can build brand love (Nguyen and Feng, 2021).

From a practical point of view, this study illustrates that practitioners in the workplace should focus on the green concept and other elements related to sustainability in order to increase EBBE. So it will provide twofold benefits, in case of environmental safety and employee positive behavior at the workplace. Finally, this study has investigated the role of three dimensions of design perception in promoting the green concept and positive behaviors, which can be termed as the contribution of the study.

## Theory and Hypotheses

The basics of design are perception and are described as a manner of looking at, perceiving, or interpreting a product. Because design necessitates creativity and imagination, perception is crucial to the business. However, perception is the starting point for creativity and imagination. Learning to see is the beginning of creation, as perception is at the heart of all creativity. Designers and marketers should be aware; however, that what one can see is more than what eyes and ears transmit to the brain. It is a result of one’s own brain. Perception is not the same as vision. The concept of vision is solid. It keeps an eye on things. The concept of perception is more abstract. Perception progresses from observation to evaluation. However, a person sees what he sees, but he perceives what he sees as a mix of what he sees, his previous experiences, and his unique perspective on a situation (Khongprakob and Kantathanawat, 2021).

According to Huh (2016), visual thinking (VT), which focuses on the use of visual pictures to link ideas, is a useful creative strategy. Intuitive, non-rational, and unsystematic thinking are all characteristics of VT. According to studies, visuals are the most important component in thinking; hence, VT is an individual’s primary way of cognition. VT has also been defined as “an active problem-solving process” (Goldschmidt, 1994), with an analytical process of observing, analyzing, and creating visual signals, as well as an interaction between seeing, picturing, and drawing (Huh, 2016).

Design is a significant strategic instrument, as evidenced by a number of studies that show how it may improve product competitiveness (Utterback, 1994). As a result, it is reasonable to conclude that in order to achieve such an advantage, designers must establish a strong brand relationship with users. Perception of visual design is a key factor in experiencing value. The external look of things translates into perceived usefulness *via* subtle clues concerning ease of use, which leads to the creation of attitudes about usability (Creusen and Schoormans, 2005). Furthermore, the visual design conveys a symbolic significance that influences aesthetic judgment, allowing the owner to utilize the object as a means of self-expression.

For most developing countries, entrepreneurship and innovation constitute the bedrock of economic success and long-term viability (Mahmud et al., 2017). This is especially true in Thailand, where the worldwide COVID-19 epidemic has wiped off conventional avenues for productivity and development (such as tourism, auto exports, and electronics manufacturing), produced a highly unpredictable labor market, and has expedited the coming of the future of work (Yingfei et al., 2021). Furthermore, multiple worldwide studies have highlighted the importance of the 21st-century workforce’s requirement for critical thinking and creative thinking abilities (Changwong et al., 2018). As a result, solutions and methods must be created to continue to foster the critical thinking abilities of tomorrow’s workforce in the “New Normal,” where a new teaching and learning process must be researched, developed, and implemented. It must also fit within the cultural and technical frameworks of their different institutions and economies as the New Normal (Moto et al., 2018).

The benefits of the functional design are studied extensively in the literature, most notably as utilitarian/functional benefits (Petruzzellis, 2010). It refers to a product’s physical attributes, including features, performance, dependability, and durability, that meet a user’s sanitary needs and expectations. These intangible elements are concealed from view by users, silently operating in the background to improve the user experience. From a means–end viewpoint, a product’s functional benefits are the bare minimum that it must provide before being supplemented with other design aspects to meet higher-order requirements. These intangible elements are concealed from view by users, silently operating in the background to improve the user experience (Avotra et al., 2021). From a means-end viewpoint, a product’s functional benefits are the bare minimum that it must provide before being supplemented with other design aspects to meet higher-order requirements.

### Brand Based Equity

The concept of creating an employer brand that exists in a different identity from the external brand yet is consistent, is flawed. Employees do not reside in a bubble that allows them to distinguish between external and internally generated information. The ambition to create “a recognizable and unique employer identity” in the minds of current and future employees must be examined in the context of existing, externally formed brand knowledge systems (Backhaus and Tikoo, 2004; Esch et al., 2006).

King and Grace (2005) differentiated the notion of brand-based equity from customer-based. For customers to see the value and, as a result, demonstrate good consumption habits, organizations must make the brand meaningful and relevant. They must also make the brand meaningful and relevant for workers to perceive value in order for them to engage in good work-related behaviors, which will result in increased brand equity. From the standpoint of the consumer, the organization aspires to foster long-term purchasing habits. In contrast, the behavior is work-related conduct centered on delivering on the brand promise from the employee’s perspective. The reality is that the information source from which these behaviors come is the same, regardless of how they emerge. As a result, EBBE is less concerned with the establishment of a brand identity, which is something that corporations do as a matter of course in their quest for competitive advantage. Rather, EBBE involves the translation of the brand identity in a form that is relevant to the employee in the context of their tasks and responsibilities, which may be described as the differential influence that brand knowledge has on an employee’s attitude to their work environment (King and Grace, 2005).

In today’s competitive environment, businesses continue to battle to gain a competitive advantage. A shift in thought has occurred toward the new dominant logic that development, survival, and competitive advantage may be accomplished not just by depending on customers, but also by appreciating the value and connection of workers. Therefore, the new problem for marketers is the inherent difficulty of recreating something that is mostly delivered as a result of human capital, namely, workers (Sundaram and Webster, 2000). The capacity to demonstrate significant amounts of brand equity is what distinguishes successful brands. High brand equity, according to Papasolomou and Vrontis (2006), is visible when firms demonstrate qualities and eventually employee perceptions that cause to produce and strengthen the level of connection, affiliation, and loyalty with organizations. Brands, according to de Chernatony and Segal-Horn (2001) and Christodoulides and de Chernatony (2010), are a sum of functional and emotional traits. To put it another way, a brand blends practical and emotional values in order to make a promise about the brand experience. As a result, a brand’s performance is determined by how successfully it delivers on its promises, which ultimately shapes employee impressions.

King and Grace (2005) describe the notion of EBBE, in particular, taking into account the specific relationship that exists between the individual and the business. Internal brand management’s purpose is to influence employee behavior in order to achieve the organization’s brand promise, but doing so successfully requires more work on the part of marketers than merely providing employees with brand-related information. While traditional organizational structures assign human resource management (HRM) to the human resources department, there has been a growing focus on employee management having a balanced approach.

Luffarelli et al. (2019) identify the literary aspects of visuals as symmetrical/direct aspects and asymmetrical/indirect aspects. They further endorsed that asymmetrical visuals can be more arousal than symmetrical visuals. This pattern has been attributed to the fact that indirect and unbalanced visuals create more meaning for individuals. Hence, following this stream present research conceptualized the phenomenon with respect to employees’ attachment and identification. Hence, it is hypnotized as:

**H1:** Visual Design perception has positive impact on Brand based equity.

Another aspect of design perception is functional. Employee views of HR procedures have a major role in affecting the success of these policies, according to HRM researchers (Takeuchi et al., 2009; Jiang et al., 2012). Through the perspective of attribution theory, Hewett et al. (2018) provided a synopsis of functional perception studies. HR procedures serve as a means of communication between employers and employees. HR practices send specific messages to employees, whether on purpose or by mistake (Bowen and Ostroff, 2004). Messages can be included in HR material (the “what”) or the implementation of HR policies (the “how”). Employee HR perceptions, according to this logic, encompass the signals workers receive from their employers as a result of watching or experiencing HR procedures. Hence, it is hypnotized as:

**H2:** Functional Design perception has positive impact on Brand based equity.

Another aspect of design perception is kinesthetic. Design that is kinesthetic in the literature on ergonomics and even human factors, kinesthetic design has been explored repeatedly (Maurya and Agarwal, 2018). These terms allude to product features such as weight, texture, form, and affordances, all of which have a significant impact on the product’s ability to be used in a pleasant, safe, straightforward, and intuitive manner. Kinesthetic design, according to Girvin et al. (2000), is an intrinsic representation of effective branding techniques, making it an important product element that impacts overall design and brand perceptions. Hence, it is hypnotized as:

**H3:** Kinesthetic Design perception has positive impact on Brand based equity.

### Green Concept

Human considerations are becoming increasingly important in the creation of green buildings. Occupant behavior, which is based on personal comfort standards, is a driving component of energy usage in office buildings (Nawaz et al., 2022). Overtime is a typical occurrence in modern society, and it poses a severe threat to energy conservation and occupant well-being (Xue et al., 2016). Because employees are the most expensive part of any business, organizations have been compelled to improve employee health, well-being, and productivity by enhancing workplace environments such as daylighting, natural ventilation, natural views, open space, places of respite, and other conveniences. The objective is to optimize health, well-being, and productivity results to be consistent with, if not improved by, the initiatives to reduce energy and resource use. Previous research has shown the links between the office environment and the health and well-being of its occupants (Lee and Maheswaran, 2011). The local surroundings and green elements of open space adjacent to the workplace influence the inhabitants’ usage habits and health perceptions, according to environmental psychology theories (Chang and Chen, 2005). The intermediating causal relationships between workers’ subjective perception design in the workplace and brand equity, on the other hand, are rarely studied.

The term “HR strength” is used frequently in the literature. Employees’ collective awareness of HR procedures is well linked to management’s aims, thanks to a solid HR system (Bowen and Ostroff, 2004). The situational strength study is where the concept of HR strength gets its start (Katou et al., 2014). Employees in a strong position have a shared awareness of the organization’s rules, practices, processes, and goals, as well as the anticipated and rewarded actions. These flexible, user-friendly policies, according to Bowen and Ostroff (2004), are green policies. Employees under a poor circumstance, on the other hand, face a great deal of ambiguity about what is expected of them in their work life, resulting in a wide range of workplace attitudes and actions (Ostrom et al., 2015). According to researchers, a strong scenario impacts employee attitudes and actions, hence, aspects of an HR system that allow for the establishment of a strong situation are crucial.

**FIGURE 1:**
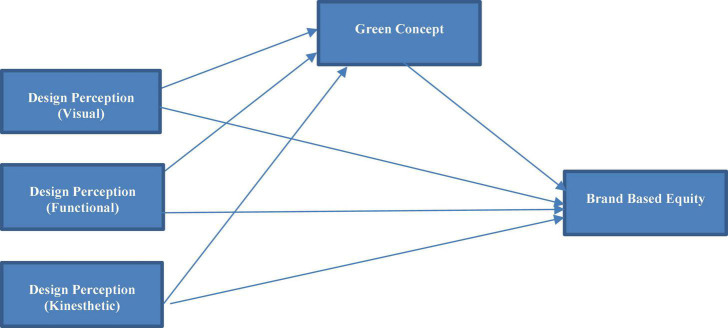
Conceptual framework.

Employee views of HR processes may be explained more fully using signaling theory. Signaling theory is concerned with strategies to use signaling activities to lessen information asymmetry between signalers, or information senders, and information receivers (Bednall et al., 2019). HR procedures are viewed as signals from management to employees in this approach. HR perception studies can be influenced by ideas on signals, signal senders, and signal receivers. Based on these notions following hypotheses of mediation relations are derived and a conceptual framework is formed based on prior literature (Figure 1).

**H4:** Green Concept mediates the relationship between Visual Design perception and Brand based equity.

**H5:** Green Concept mediates the relationship between Functional Design perception and Brand based equity.

**H6:** Green Concept mediates the relationship between Kinesthetic Design perception and Brand based equity.

## Research Methods

This study has followed a cross-sectional research design in this study to approach the study participants, and they were recruited through non-probability convenience sampling technique. Cross-sectional research design is commonly used in survey-based research. In this regard employees working in different manufacturing firms were approached where the issue of sustainability is kept under consideration by the firms such as the construction industry and mobile phone manufacturing firms. Before administrating questionnaires to the participants, prior approval from their administrator was obtained. Moreover, informed consent was obtained from the participants before asking them to complete the survey items. In addition to this, it was ensured to them that these data are being collected for academic purposes and the issue of anonymity will be kept under consideration.

With the approval from both, the participants and their administration, the survey was started and participants were offered a movie ticket in return for their response so that their motivation of the participants could be boosted. Different benchmarks were scrutinized to select a suitable number of respondents for data collection, but this study followed the criteria recommended by Krejcie and Morgan (1970) and a sample size of 384 was considered sufficient in this regard. This criterion has been used by several researchers in the past (Bashir et al., 2019, 2020; Wu et al., 2022).

Based on this criterion a sum of distributed to 400 questionnaires through personal contacts, out of which 360 were received back. After discarding the incomplete and partially filled responses the useable responses were 346. Returned questionnaires were checked for missing values and responses where missing values were higher than 5% were discarded. This sample size indicates an adequate level because this study employed Smart PLS software which handles the small sample size very comfortably (Xiaolong et al., 2021; Dar et al., 2022).

Due to the cross-sectional nature, the issue of common method biasness was likely to shatter the results, this issue was managed by following various measures. Firstly this study used reverse coded questions in the survey items to reduce the monotonic response from the participants. Secondly, it was opted to place the questions related to the variables at a different place, thus restricting the participants to generate an autocorrelation based on perception. Thirdly, we used a single-factor test to check the variance being explained by the factors, and not a single variable was explaining more than the 50% variance, confirming that there is no issue of common method biases (Podsakoff et al., 2003; Malhotra et al., 2006).

### Demographic Characteristics

Initially, the respondents were asked to rate their demographic features based on qualification, gender, experience in the current organization, and total experience. Educational level indicates that most of the participants have 16 years of education with 70% proportion in the participants while both male and female participants share almost equal portion such as [i.e., 52% (male) and 48% (female)]. A total of 50% of the employees have more than 5 years experience in their current employer while the remaining have less than 5 years experience with their current employer. While in the case of the total length of experience/service all of the respondents have more than 5 years of experience.

### Instrument Development

Responses were obtained on a 5-point Likert scale ranging from 1 to 5, where 1 indicates strongly disagree and 5 indicates strongly agree. Three dimensions of design perception, namely, visual, functional, and kinesthetic have been measured through the scale items developed by Mishra et al. (2015) and recently used by Meng and Bari (2019). For this purpose total of 15 items have been used such as “the styling of manufactured products is elegant.” This scale was partially amended with the context of the industry and participants and the wording of the statements was modified keeping in view the study background. However, the original meanings of the items that remained were not changed. The scale items indicate a good level of Cronbach’s alpha which was more than 0.60. Similarly, the second dimension of the design perception, i.e., functional is measured through seven items scale developed by Mishra et al. (2015) and recently used by Meng and Bari (2019). Sample items include “our 3D printed products offers the right number of basic features that I need.” Cronbach’s alpha value was higher than 0.60 indicating a satisfactory level. This scale was partially amended with the context of the industry and participants and the wording of the statements was modified keeping in view the study background. However, the original meanings of the items that remained were not changed. Similarly, the third dimension of design perception (kinesthetic) was measured through three items scale developed by Mishra et al. (2015) and recently used by Meng and Bari (2019). Sample item includes, “the size of products makes it easy to carry and move around.” Cronbach’s alpha value in this regard was more than 0.60 indicating a satisfactory level. This scale was partially amended with the context of the industry and participants and the wording of the statements was modified keeping in view the study background. However, the original meanings of the items remained were not changed.

The mediating variable in this study, i.e., green concept is measured through six items scale developed by Rezai et al. (2013). A sample item for this scale includes, “It makes me feel good to go green because it benefits the society.” Cronbach’s alpha value in this regard was more than 0.60 indicating a satisfactory level. The outcome [dependent variable in this study is measured through five items scale, developed by Baumgarth and Schmidt (2010)] sample item includes, “I am aware that everything I say or do can affect the brand image.”

## Results

### Assessment of Measurement and Structural Model

This study used a multivariate data analysis tool in order to test the hypotheses through structural equation modeling (SEM). For this purpose, the most commonly used partial least square (PLS) approach through Smart PLS was used. This software deals very well with the complex nature of research frameworks/models (Hair et al., 2017). Moreover, Smart PLS does not consider the distributional assumptions with regard to normality and the issue of non-normal data is dealt with comfortably. Moreover, a small sample size can also be tested through Smart PLS very comfortably and even a sample size of 49 observations can be tested easily. Assessment of SEM is based on two approaches/methods, the first one is based on a measurement model while the second one is based on a structural model (Hair et al., 2019).

Table 1 depicts the reliability and validity of the study constructs with regard to the measurement of the model. Both reliability and validity indicators have been found fit in this study, the first measure in this regard related to reliability is Cronbach’s alpha. The minimum acceptable value for this indicator of reliability is 0.60, while in this study, the alpha values have been found statistically fit, indicating a satisfactory level. The first dimension of design perception, i.e., design perception visual indicates an alpha value of 0.849, while, the alpha value of design perception functional is 0.896 and for design perception, the Kinesthetic alpha value is 0.847. The alpha value for the green concept is 0.882 and for brand-based equity it has been observed as 0.759. Similarly, another measure of reliability, i.e., rho-A has been found fit and all the values were within the acceptable range, 0.791–0.895. While the third indicator of reliability is also found to fit and values related to composite reliability were also within an acceptable range (more than 0.60), thus indicating a satisfactory level of reliability. While talking about convergent validity, we have used two measures to assess convergent validity, the first is average variance extracted (AVE) and the other is outer loadings. It has been found that the AVE of the respective constructs is greater than the threshold limits of the acceptable range (>0.50). All the constructs indicated that their AVE values were greater than the threshold value (>0.50) (Mela and Kopalle, 2002). AVE values range from 0.571 to 0.829.

**TABLE 1:** Reliability and convergent validity of the study constructs.

Construct	Item	Outer loadings	VIF	Alpha	rho-A	Composite reliability	AVE
DPF	DPF1	0.804	4.395	0.849	0.860	0.888	0.571
	DPF2	0.766	1.811				
	DPF4	0.646	1.407				
	DPF5	0.829	2.920				
	DPF6	0.755	3.922				
	DPF7	0.722	2.645				
DPK	DPK1	0.891	2.402	0.896	0.897	0.935	0.829
	DPK2	0.940	3.918				
	DPK4	0.899	2.919				
	DPV1	0.796	4.385				
DPV	DPV3	0.833	4.766	0.847	0.877	0.894	0.677
	DPV4	0.826	1.934				
	DPV5	0.837	1.801				
EBBE	EBBE1	0.653	1.262	0.759	0.791	0.845	0.579
	EBBE2	0.755	2.118				
	EBBE3	0.812	1.449				
	EBBE5	0.813	2.348				
GC	GC1	0.787	1.935	0.882	0.895	0.911	0.634
	GC2	0.885	3.710				
	GC3	0.801	2.075				
	GC4	0.690	1.529				
	GC5	0.880	3.451				
	GC6	0.712	1.697				

*DPF, design perception functional; DP, design perception kinesthetic; DPV, design perception visual; EBBE, employee-brand based equity; GC, green concept.*

**FIGURE 2:**
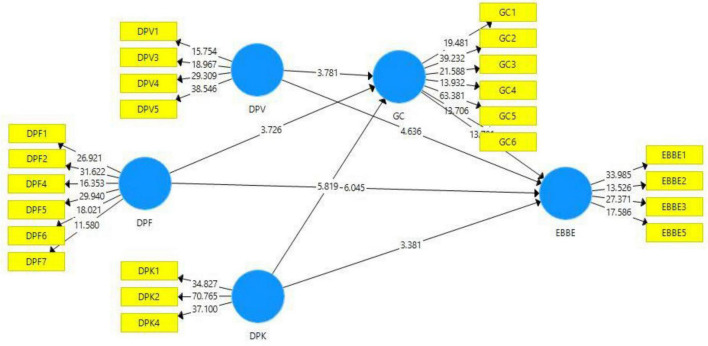
Path estimates and outer loadings.

While outer loadings were assessed in the second instance to assess the convergent validity (Figure 2 and Table 1 illustrate convergent validity). All the scale items were observed for outer loadings, and it was observed that all the scale items show a good level related to the outer loadings. Items with poor outer loadings were dropped from the analysis. In this regard, one item from the design perception functional was dropped owing to poor or weak outer loading (DPF-3), while all other items have an acceptable level of outer loadings (>0.708). Similarly, one item (DPV-2) from the dimension design perception visual has been dropped due to a weak outer loading value. No item has been dropped from design perception kinesthetic. While in the case of mediating variable, all the items indicate sufficient outer loading level, and no item was dropped. However, one item from the outcome variable (EBBE-4) was dropped due to poor outer loadings. Despite one item EBBE-1 having a lower value (less than threshold) it was retained because the AVE of this construct was within the acceptable range. Similarly, one item from the construct green concept (GC-4) was retained due to poor outer loadings, however, the AVE of the respective construct was higher than 50%.

Another dimension of validity, discriminant validity is assessed through two well-established criteria, i.e., Fornerl-Larker (1981) and HTMT ratios (Hair et al., 2017). Table 2 (Fornell-Larcker criteria) and Table 3 (HTMT ratios) in this regard illustrate discriminant value. The first criteria in this regard indicate that the square root of the AVE of variables is higher than the correlations among them (Hair et al., 2011; Bashir et al., 2020) as indicated by values in bold and underlined values reported in diagonals.

**TABLE 2:** Discriminant validity (Fornell-Larcker-1981 criteria).

Construct	DPF	DPK	DPV	EBBE	GC
DPF	*0.756*				
DPK	0.258	*0.910*			
DPV	0.431	0.191	*0.823*		
EBBE	0.547	0.385	0.535	*0.761*	
GC	0.323	0.361	0.328	0.597	*0.796*

*DPF, design perception functional; DP, design perception kinesthetic; DPV, design perception visual; EBBE, employee-brand based equity; GC, green concept.*

**TABLE 3:** Discriminant validity (HTMT).

Construct	DPF	DPK	DPV	EBBE	GC
DPF	–	–	–	–	–
DPK	0.284	–	–	–	–
DPV	0.494	0.217	–	–	–
EBBE	0.646	0.468	0.596	–	–
GC	0.358	0.401	0.362	0.740	–

*DPF, design perception functional; DP, design perception kinesthetic; DPV, design perception visual; EBBE, employee-brand based equity; GC, green concept.*

HTMT is used as a second measure to assess the discriminant validity. Two criteria were observed in this regard (liberal and conservative). Both the criteria were met as the values of HTMT ratios in all columns are less than 0.90 and 0.85, describing that both liberal and conservative criteria are met. Liberal criteria HTMT ratio indicates that the value of HTMT should not be higher than 0.90 while conservative criteria indicate that the value of HTMT should not be higher than 0.85. Table 4 illustrates the discriminant validity through HTMT ratios.

**TABLE 4:** Direct, indirect, and total path estimates.

	Beta	SD	*t*	*p*
**Direct path**				
DPF → EBBE	0.239	0.039	6.045	0.000
DPF → GC	0.166	0.045	3.726	0.000
DPK → EBBE	0.116	0.034	3.381	0.001
DPK → GC	0.283	0.049	5.819	0.000
DPV → EBBE	0.214	0.046	4.636	0.000
DPV → GC	0.201	0.053	3.781	0.000
GC → EBBE	0.515	0.038	13.701	0.000
**Indirect path**				
DPF → GC → EBBE	0.086	0.023	3.653	0.000
DPK → GC → EBBE	0.146	0.026	5.560	0.000
DPV → GC → EBBE	0.103	0.028	3.674	0.000
**Total path**				
DPF → EBBE	0.324	0.042	7.766	0.000
DPK → EBBE	0.262	0.038	6.922	0.000
DPV → EBBE	0.317	0.053	5.969	0.000

*DPF, design perception functional; DP, design perception kinesthetic; DPV, design perception visual; EBBE, employee-brand based equity; GC, green concept.*

Model fitness was assessed through the coefficient of determination (*R*^2^) and effect size (*f*^2^). Table 4 illustrates the quality criteria based on the coefficient of determination. Here, it has been observed that predictors along with the green concept are explaining 45% variation in the EBBE, indicating a good level of variance. While three dimensions of design perception were explaining 21% of perception in the green concept (mediating variable) thus predicting a good and reasonable model fitness (Bashir et al., 2020), and a satisfactory level (Hair et al., 2017). In this study, we have also assessed the predictive relevance of the model through *Q*^2^ (Geisser, 1975), and it has been observed that the value of *Q*^2^ was higher than zero, which indicates a good model predictive relevance.

### Hypotheses Testing

The last stage of assessment of the structural model is related to path estimates among study constructs and hypotheses testing. Table 4 in this regard illustrates direct, indirect, and total path estimates, while Table 5 illustrates hypotheses testing. The first hypothesis of this study is related to visual design perception and brand based equity. Statistical data indicates that the impact of visual design perception is positive on EBBE (*b* = 0.214, *t* = 4.636, and *p* = 0.000). The coefficient value indicates that positive perception regarding visual design triggers employees to develop brand-based equity (H1 supported). These findings are in connection with the previous studies as noted by King and Grace (2005) that EBBE, in particular, takes into account the specific relationship that exists between the individual and the business. Internal brand management’s purpose is to influence employee behavior in order to achieve the organization’s brand promise, while traditional organizational structures assign HRM to the human resources department, there has been a growing focus on employee management having a balanced approach.

**TABLE 5:** Hypotheses testing.

		Coefficient (beta)	SD	*t*	*p*	Status
**Hypotheses**						
H1	DPV → EBBE	0.214	0.046	4.636	0.000	Supported
H2	DPF → EBBE	0.239	0.039	6.045	0.000	Supported
H3	DPK → EBBE	0.116	0.034	3.381	0.001	Supported
**Mediation hypotheses**						
H4	DPV → GC → EBBE	0.103	0.028	3.674	0.000	Supported
H5	DPF → GC → EBBE	0.086	0.023	3.653	0.000	Supported
H6	DPK → GC → EBBE	0.146	0.030	5.56	0.000	Supported

*DPF, design perception functional; DP, design perception kinesthetic; DPV, design perception visual; EBBE, employee-brand based equity; GC, green concept.*

Luffarelli et al. (2019) identify the literary aspects of visuals as symmetrical/direct aspects and asymmetrical/indirect aspects. They further endorsed that asymmetrical visuals can be more arousal than symmetrical visuals. This pattern has been attributed to the fact that indirect and unbalanced visuals create more meaning for individuals.

The second hypothesis of this study is related to the impact of functional design perception and brand-based equity. Results indicate that the impact of functional design perception is positive on EBBE (*b* = 0.239, *t* = 6.045, and *p* = 0.000). The coefficient value indicates that positive perception regarding functional elements triggers employees to develop brand-based equity (H2 supported). These findings are in connection with the previous studies done by HRM researchers (Takeuchi et al., 2009; Jiang et al., 2012). Through the perspective of attribution theory, Hewett et al. (2018) provided a synopsis of functional perception studies. HR procedures serve as a means of communication between employers and employees. HR practices send specific messages to employees, whether on purpose or by mistake (Bowen and Ostroff, 2004).

The third hypothesis of this study is related to kinesthetic design perception and brand-based equity. Statistical data indicates that the impact of kinesthetic design perception is positive on EBBE (*b* = 0.116, *t* = 3.381, and *p* = 0.000). The coefficient value indicates that positive perception regarding the kinesthetic element of design perception triggers employees to develop brand-based equity (H3 supported). These findings indicate that design kinesthetic in the literature kinesthetic design has been explored repeatedly (Maurya and Agarwal, 2018). These terms allude to product features such as weight, texture, form, and affordances, and indicate that it has a pleasant, safe, straightforward, and intuitive impact on employees’ perception. Kinesthetic design, according to Girvin et al. (2000), is an intrinsic representation of effective branding techniques, making it an important product element that impacts overall design and brand perceptions.

Similarly, the indirect effect (H4) for the path DPV → GC → EBBE, has been found statistically significant (*p* < 0.05) (H4 supported). For other two mediating paths, DPF → GC → EBBE (H5) and DPK → GC → EBBES (H6) have been found statistically significant (*p* < 0.05) (H5 and H6 supported). All these mediating paths are tested on the basis of indirect effect and their significance level. These findings support the argument green concept can trigger more positive emotions and employee can develop brand-based equity. Human considerations are becoming increasingly important in the creation of green buildings. Occupant behavior, which is based on personal comfort standards, is a driving component of energy usage in office buildings. Overtime is a typical occurrence in modern society, and it poses a severe threat to energy conservation and occupant well-being (Xue et al., 2016).

Owning to the reason that employees are very crucial for business, organizations have been compelled to improve employee health, well-being, and productivity by enhancing workplace environments such as daylighting, natural ventilation, natural views, open space, places of respite, and other conveniences. The objective to optimize health, well-being, and productivity results are consistent with, if not improved by, initiatives to reduce energy and resource use. Previous research has shown the links between the office environment and the health and well-being of its occupants (Lee and Maheswaran, 2011).

The term “HR strength” is used frequently in the literature. Employees’ collective awareness of HR procedures is well linked to management’s aims, thanks to a solid HR system (Bowen and Ostroff, 2004). The situational strength study is where the concept of HR strength gets its start (Katou et al., 2014). Employees in a strong position have a shared awareness of the organization’s rules, practices, processes, and goals, as well as the anticipated and rewarded actions. These flexible, user-friendly policies, according to Bowen and Ostroff (2004), are green policies. Employees in poor circumstances, on the other hand, face a great deal of ambiguity about what is expected of them in their work life, resulting in a wide range of workplace attitudes and actions (Ostrom et al., 2015).

## Conclusion

On the basis of empirical evidence, it can be safely concluded that the impact of visual design perception is positive on EBBE and a positive evaluation of visual design motivates employees to develop brand based equity. EBBE, in particular, takes into account the specific relationship that exists between the individual and the business. Internal brand management can influence employee behavior in order to achieve the organization’s brand promise. This pattern has been attributed to the fact that indirect and unbalanced visuals create more meaning for individuals. Moreover, functional design perception is positive on EBBE and functional elements trigger employees to develop brand-based equity. Moreover, kinesthetic design perception is positive on EBBE, and design kinesthetic depicts that weight, texture, form, and affordances, have a pleasant, safe, straightforward, and intuitive impact on employees’ perception.

## Theoretical and Practical Implications

From a theoretical perspective, this study is the first to document the impact of the element of design perception on EBBE. This study adds to the present stream of the brand-based equity research and contends that design elements of products in the manufacturing industry can influence the employees’ perception and it can foster positive behavior among employees. So this study can be termed the first study which has examined the relationship between design perception elements and EBBE. Secondly, this study tested the mediating role of the green concept, which is another contribution of this study. This study tends to advocate that the green concept can increase EBBE. From a practical point of view, this study illustrates that practitioners in the workplace should focus on the green concept and other elements related to sustainability in order to increase EBBE. So it will provide twofold benefits, in case of environmental safety and employee positive behavior.

## Limitations and Future Directions of the Study

This study has also some potential limitations; first, it is a cross-sectional study and does not permit drawing a causal inference in this regard. Second, data are collected from only manufacturing firms which can also be expanded in future studies. Moreover, this study only anticipated the green concept as a mediating variable, while in future studies other potential mediating and moderating mechanisms can also be tested. In this regard, green behavior, and job satisfaction can be tested in future studies. In addition to this safety, eldership can also be tested as a moderating phenomenon in the future. This study tested mediation through the indirect effects, and no specification i.e. either partial or full mediation was observed, thus, in the future study, the author can also inspect the nature of mediation too. Moreover, collecting a larger sample can provide important and deeper insights in this regard. In the future researchers can opt to investigate and explore the impact of design perception in shaping brand love in luxury products designs/manufacturers.

## Data Availability Statement

The original contributions presented in the study are included in the article/supplementary material, further inquiries can be directed to the corresponding author.

## Author Contributions

YL conceived and designed the concept and wrote the manuscript. The author read and agreed to the published version of the manuscript.

## Conflict of Interest

The author declares that the research was conducted in the absence of any commercial or financial relationships that could be construed as a potential conflict of interest.

## Publisher’s Note

All claims expressed in this article are solely those of the authors and do not necessarily represent those of their affiliated organizations, or those of the publisher, the editors and the reviewers. Any product that may be evaluated in this article, or claim that may be made by its manufacturer, is not guaranteed or endorsed by the publisher.
